# Supplementary material for: Changes in inflammatory gene expression in brain tissue adjacent and distant to a viable cyst in a rat model for neurocysticercosis
Source: PLoS Negl Trop Dis. 2021 Apr 27;15(4):e0009295. doi: 10.1371/journal.pntd.0009295 (PMC8104410; doi:10.1371/journal.pntd.0009295)
Supplement: S1 Table — Tissue close to the parasite (CP), contralateral tissue (CT) and non-infected tissue were collected (N) from different brain areas anterior (A), medium (M) and posterior (P). # Cysts refers to the number of cysts found in each rat and P cysts refers to the number of cysts located in the parenchymal tissue. (DOCX) [file pntd.0009295.s001.docx]

| Rat  Code | #  Cysts | P  cysts | Location |
| --- | --- | --- | --- |
| R07 | 2 | 1 | CP-A |
| R08 | 2 | 1 | CP-A |
| R09 | 4 | 2 | CP-A |
| R10 | 2 | 1 | CP-A |
| R11 | 2 | 1 | CP-A |
| R12 | 2 | 1 | CP-A |
| R13 | 1 | 1 | CP-A |
| R14 | 2 | 1 | CP-A |
| R15 | 3 | 1 | CP-M |
| R16 | 2 | 2 | CP-P |
| R17 | 1 | 1 | CP-P |
| R18 | 1 | 1 | CP-P |
| R19 | 2 | 1 | CP-P |
| R07 | 2 | 1 | CT-A |
| R08 | 2 | 1 | CT-A |
| R09 | 4 | 2 | CT-A |
| R10 | 2 | 1 | CT-A |
| R11 | 2 | 1 | CT-A |
| R12 | 2 | 1 | CT-A |
| R13 | 1 | 1 | CT-A |
| R14 | 2 | 1 | CT-A |
| R15 | 3 | 1 | CT-M |
| R16 | 2 | 2 | CT-P |
| R17 | 1 | 1 | CT-P |
| R18 | 1 | 1 | CT-P |
| R19 | 2 | 1 | CT-P |
| R01 | 0 | 0 | N-A |
| R02 | 0 | 0 | N-A |
| R03 | 0 | 0 | N-A |
| R04 | 0 | 0 | N-A |
| R05 | 0 | 0 | N-A |
| R06 | 0 | 0 | N-A |
| R01 | 0 | 0 | N-P |
| R02 | 0 | 0 | N-P |
| R03 | 0 | 0 | N-P |
| R04 | 0 | 0 | N-P |
| R05 | 0 | 0 | N-P |
| R06 | 0 | 0 | N-P |

Table S1. Number of tissues used in this study. Tissue close to the parasite (CP), contralateral tissue (CT) and non-infected tissue were collected (N) from different brain areas anterior (A), medium (M) and posterior (P). *# Cysts* refers to the number of cysts found in each rat and *P cysts* refers to the number of cysts located in the parenchymal tissue.
