# Supplementary figures and images for: Changes in inflammatory gene expression in brain tissue adjacent and distant to a viable cyst in a rat model for neurocysticercosis
Source: PLoS Negl Trop Dis. 2021 Apr 27;15(4):e0009295. doi: 10.1371/journal.pntd.0009295 (PMC8104410; doi:10.1371/journal.pntd.0009295)

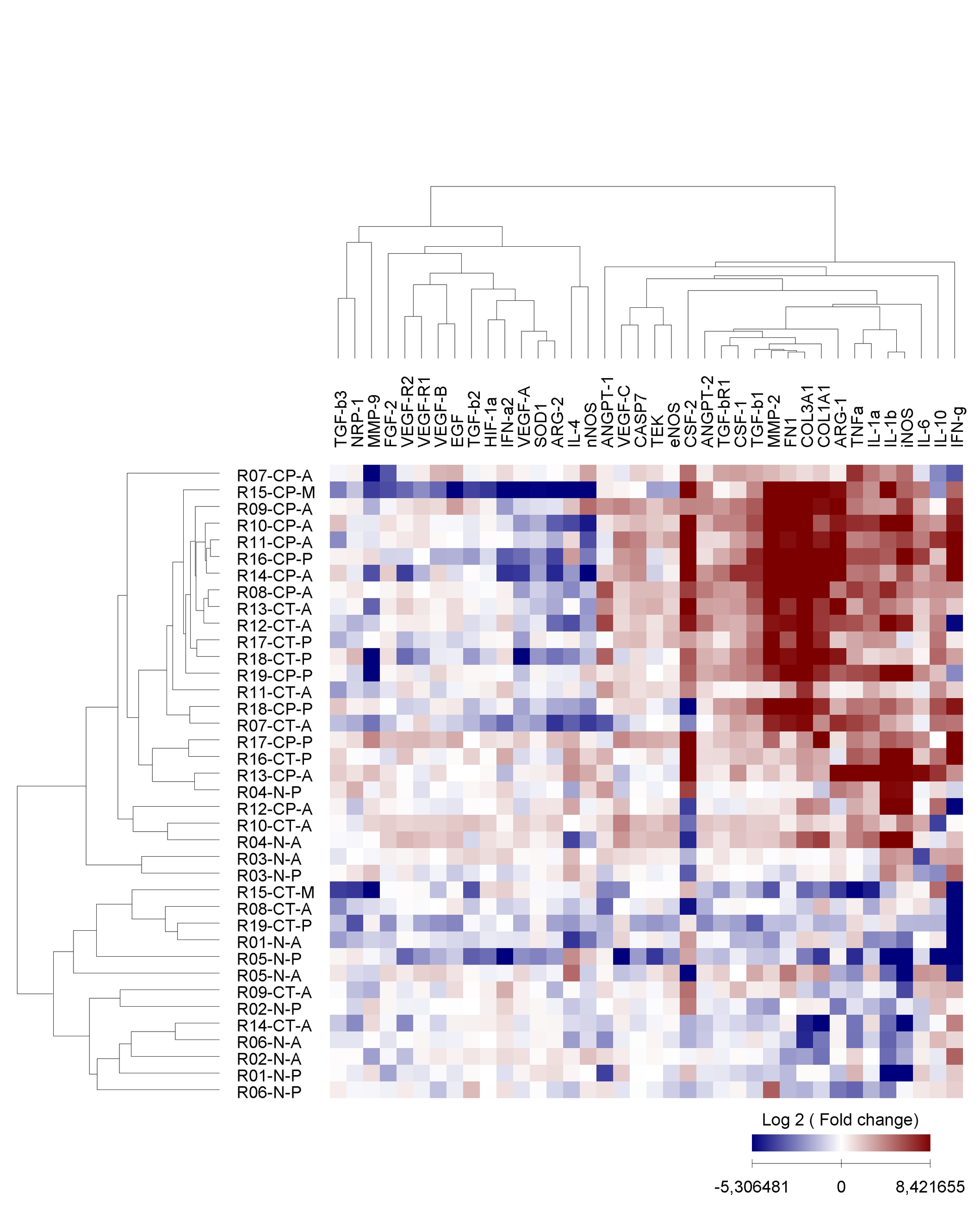

Supplement: S1 Fig — Gene responses in tissue close to the parasite (CP) form a cluster but also shared its expression with its contralateral tissue (CT). Some CT share the gene profile presented in non-infected tissue (N). Three different regions of the brain were used anterior (A), medial (M) and posterior (P). Bar represents log2 of fold change. (TIF) [file pntd.0009295.s002.tif]
