# Supplementary material for: Changes in inflammatory gene expression in brain tissue adjacent and distant to a viable cyst in a rat model for neurocysticercosis
Source: PLoS Negl Trop Dis. 2021 Apr 27;15(4):e0009295. doi: 10.1371/journal.pntd.0009295 (PMC8104410; doi:10.1371/journal.pntd.0009295)
Supplement: Attachment — Submitted filename: letter to response editors.docx [file pntd.0009295.s003.docx]

January 25, 2021

Adly M.M. Abd-Alla

Guest Editor

PLOS Neglected Tropical Diseases

Mar Siles-Lucas

Deputy Editor

PLOS Neglected Tropical Diseases

Dear Editors,

We are now returning our revised Manuscript PNTD-D-19-01926 Version 1 entitled “Changes in inflammatory gene expression in brain tissue adjacent and distant to a viable cyst in a rat model for neurocysticercosis”. We thank you and the Reviewers for all of the suggestions and corrections in our original manuscript; they have been very important and constructive for us. We hope the manuscript is now suitable for publication in the prestigious PLOS Neglected Tropical Diseases, but we will be happy to make further changes if necessary. Below is the response to editorial comments and reviewers.

**Editorial comments**

*(1) A letter containing a detailed list of your responses to the review comments and a description of the changes you have made in the manuscript.*

**- In this letter we are responding to the comments of the editors and reviewers of the manuscript.**

*(2) Two versions of the manuscript: one with either highlights or tracked changes denoting where the text has been changed (uploaded as a "Revised Article with Changes Highlighted" file ); the other a clean version (uploaded as the article file).*

**- We are attaching the two versions of the manuscript***: one with tracked changes denoting where the text has been changed (uploaded as a "Revised Article with Changes Highlighted" file ); the other a clean version (uploaded as the article file).*

*(3) If available, a striking still image (a new image if one is available or an existing one from within your manuscript). If your manuscript is accepted for publication, this image may be featured on our website. Images should ideally be high resolution, eye-catching, single panel images; where one is available, please use 'add file' at the time of resubmission and select 'striking image' as the file type.*

*Please provide a short caption, including credits, uploaded as a separate "Other" file. If your image is from someone other than yourself, please ensure that the artist has read and agreed to the terms and conditions of the Creative Commons Attribution License at http://journals.plos.org/plosntds/s/content-license (NOTE: we cannot publish copyrighted images).*

**- All images are original.**

*(4) Appropriate Figure Files*

*Please remove all name and figure # text from your figure files upon submitting your revision. Please also take this time to check that your figures are of high resolution, which will improve both the editorial review process and help expedite your manuscript's publication should it be accepted. Please note that figures must have been originally created at 300dpi or higher. Do not manually increase the resolution of your files. For instructions on how to properly obtain high quality images, please review our Figure Guidelines, with examples at:* [*http://journals.plos.org/plosntds/s/figures*](http://journals.plos.org/plosntds/s/figures)

*While revising your submission, please upload your figure files to the Preflight Analysis and Conversion Engine (PACE) digital diagnostic tool, https://pacev2.apexcovantage.com/ PACE helps ensure that figures meet PLOS requirements. To use PACE, you must first register as a user. Then, login and navigate to the UPLOAD tab, where you will find detailed instructions on how to use the tool. If you encounter any issues or have any questions when using PACE, please email us at* [*figures@plos.org*](mailto:figures@plos.org)*.*

- **All figures are correct.**

**Response to reviewers' comments**

*<b>Key Review Criteria Required for Acceptance?</b></br></br>*

*As you describe the new analyses required for acceptance, please consider the following:</br></br>*

*<b>Methods</b></br></br>*

*-Are the objectives of the study clearly articulated with a clear testable hypothesis stated?</br>*

*-Is the study design appropriate to address the stated objectives?</br>*

*-Is the population clearly described and appropriate for the hypothesis being tested?</br>*

*-Is the sample size sufficient to ensure adequate power to address the hypothesis being tested?</br>*

*-Were correct statistical analysis used to support conclusions?</br>*

*-Are there concerns about ethical or regulatory requirements being met?</br></br>*

***Reviewer #1:*** *The objectives are well articulated with a clear testable hypothesis.*

*Study design appropriate.*

*Sample size is small, but the results striking and is an animal model.*

*Statistical anaylsis appropriate*

***Reviewer #2:*** *I have no concerns regarding the methods of the study.*

*--------------------*

*<b>Results</b></br></br>*

*-Does the analysis presented match the analysis plan?</br>*

*-Are the results clearly and completely presented?</br>*

*-Are the figures (Tables, Images) of sufficient quality for clarity?</br></br>*

***Reviewer #1:*** *Analysis plan is appropriate. The results are clearly presented.*

*Figures are sufficient.*

*Page 6 - the two lines 141-142 give the results and use symbols (*,#,etc) - I found this hard to follow.*

*Page 7- 159-161 - same comments applies to page 6.*

- **Thank you for this comment, the symbols were changed for only asterisk.**

***Reviewer #2:*** *I have no concerns with the results section.*

*--------------------*

*<b>Conclusions</b></br></br>*

*-Are the conclusions supported by the data presented?</br>*

*-Are the limitations of analysis clearly described?</br>*

*-Do the authors discuss how these data can be helpful to advance our understanding of the topic under study?</br>*

*-Is public health relevance addressed?</br></br>*

***Reviewer #1:*** *The conclusions supported by the data supported and has important implications for the field of NCC. Hippocampal atrophy is an emerging field and more data is needed. The authors do a nice job of laying this out for the reader. The discussion of the results, which are very interesting, can help us understand pathogenesis hippocampal atrophy in the setting of NCC and the public health relevance is addressed.*

*The limitations is are addressed.*

*Reviewer #2: No concerns with the conclusions section.*

*--------------------*

*<b>Editorial and Data Presentation Modifications?</b></br><br/>*

*Use this section for editorial suggestions as well as relatively minor modifications of existing data that would enhance clarity. If the only modifications needed are minor and/or editorial, you may wish to recommend “Minor Revision” or “Accept”.*

***Reviewer #1:*** *Page 4 - line 73 - authors note that leading to a cascade of inflammatory responses that cause cyst degeneration - I pulled reference 6 and there is no discussion of inflammatory response.*

- **We appreciate this comment, the sentence has been clarified to mention that the cyst degeneration is accompanied by an associated immune inflammation***.*

***Reviewer #2:*** *Minor criticisms are as follows:*

*1. The sentence on lines 79-81 has a lot of commas and is confusing to read. I would separate into 2 sentences.*

**- Thank you, we have included this suggestion.**

*2. Line 231 – would add the phrase “in CT samples” to the end of the sentence, “As expected, fibrosis-related genes…..”*

**- Thank you, we clarified this sentence.**

*3. Line 246 – would change “NCC” to say “viable NCC” as the expected inflammatory profile would differ significantly in degenerating or calcified lesions.*

**- Thank you, we included this suggestion.**

*4. Line 250-251, would add that chronic seizures are also thought to lead directly to hippocampal atrophy*

**- Thank you, we included this suggestion**

*5. Lines 257-259 – between “found” and “changes” would put the word that.*

*Or could change to “this is the first report… that showed that changes”*

- **Thank you we replaced this word***.*

*6. Line 261 – “in order understand” should be changed to “in order to understand”*

**- Thank you we replaced this word.**

*--------------------*

*<b>Summary and General Comments</b></br></br>*

*Use this section to provide overall comments, discuss strengths/weaknesses of the study, novelty, significance, general execution and scholarship. You may also include additional comments for the author, including concerns about dual publication, research ethics, or publication ethics. If requesting major revision, please articulate the new experiments that are needed.*

***Reviewer #1:*** *The work presented is interesting. The hypothesis is based on clinical observations on the association of hippocampal atrophy in the setting of NCC. The authors set up a lovely experiment to begin to unravel pathogenesis. The results support that there is increased expression of inflammatory cytokines is over expressed in tissue close to the parasite - which is expected. But, genes for IL-1a, CSF-1, FN-1, COL-3A1, and MMP-2 were over expressed in contraleral tissue compared to non-infected tissue. This is quite novel and the results have clinical implications.*

***Reviewer #2:*** *The paper by Carmen-Orozco et al discusses an extremely important topic, that of the role of inflammation in the pathophysiology of NCC. The authors are the first to demonstrate an upregulation of pro-inflammatory and pro-fibrosis genes on the contra-lateral side to a viable NCC lesion. This provides extremely important information regarding the possible mechanisms by which epilepsy can develop in NCC. Overall, the paper is well written and provides significant new information that will be very useful to those studying NCC and/or epilepsy.*

*I have a few chief questions for the authors:*

*1. Did any of the infected rats experience seizures during the study? Although seizures are more common with degenerating and calcified lesions, they can still occur with viable lesions and this could be an important confounder as seizures are also known to trigger inflammation.*

**- This is an important question to address, we are writing a manuscript where we monitored infected rats by video and EEG. However, for this group of study we did not monitored the rats 24/7, and our personal who took care of the animals did not report any behavioral abnormality in the group of animals used in this study.**

*2. Line 77 – stated that the study of viable lesions can “provide pivotal information about NCC etiology and its symptoms.” I would change etiology to pathophysiology, as we already know the etiology of NCC.*

**- Thank you we replaced this word.**

*3. Methods section, line 100 – They state that for all infected rats, “the tissue surrounding the cyst was dissected and collected.” However, some of the rats had 2 cysts – for these rats, did they collect both cysts or just one? And if they collected only one, how did they chose between cysts? Also, did they do any analyses to compare those with 1 cyst to those with 2? Was there greater inflammation if there were 2 cysts?*

**- Thank you for this question. We have updated the heatmap (figure 4) pointing out the rats where 2 parenchymal cysts were found (asterisk added), in total 11 rats carrying 1 parenchymal cyst and 2 rats with 2 parenchymal cysts were used. As it is shown on the figure 4, rats carrying 2 parenchymal cysts do not show a dramatic change in gene expression. It is important to mention that rats carrying up to 4 cysts (1 rat) were used but those cysts were subarachnoid cysts and were in the same hemisphere but distal to the evaluated tissue. Additionally, no brain compression was observed. A better description for each rat was included on the supplementary table 1. We performed a spearman correlation between levels of expression and number of the cyst and no correlation was found for most of the genes were changes in expression is been reported. With exception of Angpt2 (rho=0.5647, p=0.0443) and Tgfb3 (rho=-0.5868, p=0.0350). However, when we looked for the expression profile of rats having 3 (Rat 15) and 4 cysts (Rat 09), its levels were comparable to rats having 1 or 2 cysts (Figure 4), showing that the inflammation was not apparently greater.**

*4. Discussion, lines 235-237 – The authors state that, even though they did not find increased gene expression of angiogenesis, “it is evident that in the tissue surrounding the cyst, angiogenesis is present and expected.” How was this evident that angiogenesis was present? Was this seen on pathology?*

**- Thank you for this question, we described previously the presence of angiogenesis and blood brain barrier disruption as well angiogenesis induction by *T. solium* excretory and secretory products. However here we did not find increased levels of angiogenic factors. We believe this is because of the complex VEGF mRNA processing and because of the different isoforms presented.**

*5. Figure 4 – it is impossible, looking at the figure the way it is, to compare the groups. Would separate the CP, CT, and N groups into three separate parts of the figure so you can actually compare between the groups.*

**- We appreciate this comment, we are including a different heatmap. We have separated the heatmap in three groups to illustrate the gene expression changes in the contralateral tissue. For this map, each tissue close to the parasite is next to its contralateral side.**

*PLOS authors have the option to publish the peer review history of their article (<a href="https://journals.plos.org/plosntds/s/editorial-and-peer-review-process#loc-peer-review-history" target="_blank">what does this mean?</a>). If published, this will include your full peer review and any attached files.<br><br>*

*If you choose “no”, your identity will remain anonymous but your review may still be made public.<br><br>*

*<b>Do you want your identity to be public for this peer review?</b> For information about this choice, including consent withdrawal, please see our <a href="https://www.plos.org/privacy-policy" target="_blank">Privacy Policy</a>.*

***Reviewer #1:*** *Yes: Christina coyle*

***Reviewer #2:*** *No*

I should mention that, Peru-JHU TMRC Program is being included in the *Acknowledgements* section due to the support given in the execution of the study.

The results of this study have not and will not be submitted for publication elsewhere as long as it is under consideration by PLOS Neglected Tropical Diseases.

I declare that none of the authors has a conflict of interest (financial, ethical and personal) and that the material is original and have not already been published. I have not submitted a similar manuscript for publication elsewhere. All co-authors agree with the corrections and with the new version of the manuscript.

Sincerely yours,

Manuela R. Verastegui

Professor

Universidad Peruana Cayetano Heredia

Phone (511) 4832942, mobile (51) 997521753

[Manuela.verastegui@upch.pe](mailto:Manuela.verastegui@upch.pe)
